# Point-of-care cerebrospinal fluid Gram stain for the management of acute meningitis in adults: a retrospective observational study

**DOI:** 10.1186/s12941-020-00404-9

**Published:** 2020-12-07

**Authors:** Tomohiro Taniguchi, Sanefumi Tsuha, Soichi Shiiki, Masashi Narita

**Affiliations:** 1 Division of Infectious Diseases, Department of Internal Medicine, Okinawa Chubu Hospital, 281 Miyazato, Uruma, Okinawa 904-2293 Japan; 2 Division of General Internal Medicine and Infectious Diseases, Hiroshima Prefectural Hospital, 1-5-54 Ujinakanda, Minamiku, Hiroshima 734-8530 Japan; 3 Division of General Internal Medicine and Infectious Diseases, Sakibana Hospital, 1-3-30 Nozomino, Izumi, Osaka 594-1105 Japan

**Keywords:** Point-of-care gram stain, Cerebrospinal fluid, Acute meningitis, Bacterial meningitis, Suppurative meningitis, Aseptic meningitis

## Abstract

**Background:**

Gram stain of cerebrospinal fluid (CSF) is widely used in the diagnosis of acute meningitis, however, it is often conducted in the laboratory, as only some hospitals have access to point-of-care Gram stain (PCGS). The purpose of this study was to demonstrate the clinical impact and utility of PCGS in diagnosing and treating both bacterial and aseptic meningitis in adults.

**Methods:**

This was a hospital-based, retrospective observational study at a referral center in Okinawa, Japan. We reviewed the records of all patients aged 15 years or older who were admitted to the Division of Infectious Diseases between 1995 and 2015 and finally diagnosed with bacterial (n = 34) or aseptic meningitis (n = 97). For bacterial meningitis, we compared the treatments that were actually selected based on PCGS with simulated treatments that would have been based on the Japanese guidelines. For aseptic meningitis, we compared the rates of antibiotic use between real cases where PCGS was available and real cases where it was not.

**Results:**

PCGS was the most precise predictor for differentiating between bacterial and aseptic meningitis (sensitivity 91.2%, specificity 98.9%), being superior in this regard to medical histories, vital signs and physical examinations, and laboratory data available in the emergency room (ER). In bacterial meningitis, PCGS reduced the frequency of meropenem use (1/34 = 3.0%) compared with simulated cases in which PCGS was not available (19/34 = 55.9%) (*p*< 0.001). In aseptic meningitis cases, the rate of antibiotic administration was lower when PCGS was used (38/97 = 39.2%) than when it was not (45/74 = 60.8%) (*p* = 0.006).

**Conclusions:**

PCGS of CSF distinguishes between bacterial and aseptic meningitis more accurately than other predictors available in the ER. Patients with bacterial meningitis are more likely to receive narrower-spectrum antimicrobials when PCGS is used than when it is not. PCGS of CSF thus can potentially suppress the empiric use of antimicrobials for aseptic meningitis.

## Background

Rapid diagnosis of bacterial meningitis and early antimicrobial treatment are essential for a favorable outcome [1–3], and empiric broad-spectrum antibiotics should be given as soon as possible to patients with suspected bacterial meningitis. At the same time, these broad-spectrum antibiotics should not be administered if not needed: antimicrobial-resistant meningitis, including meropenem-resistant *Streptococcus pneumoniae* in the CSF, has already been isolated, and its spread in Japan and Taiwan is a cause for concern [4, 5]. Distinguishing bacterial meningitis from aseptic meningitis, which is caused mainly by viral infection, is therefore crucial, but is challenging because the symptoms are often similar [3, 6]. There is no single laboratory test that adequately discriminates between bacterial and aseptic meningitis [7, 8]. Due to this diagnostic uncertainty, most patients with aseptic meningitis are unnecessarily treated with broad-spectrum antibiotics [3].

Gram stain of the cerebrospinal fluid (CSF) is a rapid, inexpensive, and accurate method for detecting the etiologic agents of bacterial meningitis [6, 9]. The sensitivity of CSF Gram staining is 60–98%, depending on the patient population, type of bacteria, and previous antimicrobial treatment, and its specificity is nearly 100% [9–17]. Current guidelines from the Infectious Diseases Society of America recommend Gram staining of the CSF for all suspected meningitis patients [8]; in practice, however, this is only possible at the select institutions where Gram stain examination by a microbiology technician is available on demand at any time. At institutions where Gram staining is not available around the clock, empiric antibiotics are started in 60.8% (45/74) of aseptic meningitis cases in adults [11].

At Okinawa Chubu Hospital in Japan, point-of-care Gram stain (PCGS) has been used routinely for the diagnosis of infectious diseases since 1976 [18]. As PCGS can be performed by clinicians in the emergency room (ER) at any time, our physicians rely on PCGS to help them choose appropriate antibiotics. However, the utility of PCGS in treating acute meningitis has never been evaluated, and its clinical impact remains unclear.

The objective of this study was to clarify whether PCGS performed by clinicians is a valuable tool for the diagnosis and treatment of bacterial and aseptic meningitis in adults. For that purpose, we evaluated whether PCGS can discriminate between bacterial and aseptic meningitis, whether the selection of targeted therapies based on PCGS results as opposed to reliance on the Japanese guidelines alone can suppress broad-spectrum antimicrobial use for bacterial meningitis, and whether PCGS as opposed to no Gram stain findings can limit the use of empiric antibiotics for aseptic meningitis. Our findings lead us to recommend PCGS to ensure a more precise diagnosis of acute meningitis and to reduce the overuse of broad-spectrum antimicrobial agents for both bacterial and aseptic meningitis.

## Methods

### Study design and setting

This was a hospital-based, retrospective observational study. The study setting was Okinawa Chubu Hospital, located in central Okinawa, a subtropical region of Japan. Approximately 39,000 patients visit the ER annually and nearly 7,000 patients are hospitalized through the ER each year [19]. Most patients who are suspected of meningitis are initially worked up at the ER. Adolescent and adult patients who are diagnosed with bacterial or aseptic meningitis are admitted to the Division of Infectious Diseases, while patients with meningitis related to surgical interventions such as ventricular peritoneal shunt infection are admitted to the Division of Neurosurgery and patients with encephalitis are admitted to the Division of Neurology.

### Case definitions and data collection

Bacterial meningitis was defined as clinically evident acute meningitis and positive routine bacterial culture of CSF, regardless of whether antibiotics were administered before the first CSF sample was obtained. This definition was used because bacterial culture is still regarded as the gold standard for the diagnosis of bacterial meningitis [20, 21]. Aseptic meningitis was defined as clinically evident acute meningitis with pleocytosis in the CSF and no growth on routine bacterial culture of the CSF [16, 22].

In this study, all clinical information of acute meningitis was collected from hospital database between January 1995 and January 2015. The inclusion criteria were (1) all patients aged 15 years or older who were (2) admitted to the Division of Infectious Diseases and who were (3) finally diagnosed with bacterial or aseptic meningitis. The exclusion criteria were (1) bacterial meningitis in which CSF culture was negative, a situation that was typically caused by prior antimicrobial exposure, which we chose to exclude because this study was intended to investigate culture-proven bacterial meningitis; (2) certain pathogens such as *Bartonella*, *Leptospira*, and *Mycobacterium tuberculosis* that were not incubated in routine CSF cultures; (3) aseptic meningitis in which prior antimicrobial exposure was positive within 48 h before the first CSF sample was obtained and also in which empiric antimicrobials were started after CSF sampling (this qualification was intended to exclude false culture-negative bacterial meningitis); (4) aseptic meningitis caused by non-infectious diseases such as Sjögren's syndrome, which we chose to exclude because this study was focused on infectious meningitis; (5) all cases in human immunodeficiency virus (HIV)-infected patients, which we chose to exclude because their opportunistic infections and clinical courses are quite different from those in cases of community-acquired meningitis; (6) meningitis cases that had been diagnosed at a previous hospital and referred to our hospital; and (7) cases whose CSF data was missing.

### CSF examinations

CSF specimens obtained in the ER were immediately transported to a laboratory where conventional tests including cell counts, glucose and protein levels were measured for every patient. Each sample was first centrifuged at 1500 rpm for 5 min. As soon as the supernatant had been discarded, the sediment was returned to the ER for PCGS. All CSF sediment samples were placed on glass slides, fixed with flame or hot air, and Gram stained with Barmii M manufactured by Muto Pure Chemicals (Tokyo, Japan). We used a four-step of dying procedure using crystal violet, 2% iodine sodium hydroxide, acetone ethyl alcohol, and 0.1% fuchsine. The slides were examined in the ER by in-house staff members including trained resident physicians (postgraduate years 1 and 2) to identify each etiologic agent and select an appropriate targeted antimicrobial therapy [18, 23]. Staff members examined the slides under conventional light microscopy at 100 × magnification, then at 1000 × magnification under immersion oil to semi-quantify CSF polymorphonuclear neutrophils or mononuclear lymphocytes and to search for bacteria or yeasts. All findings were confirmed by senior residents (postgraduate years 3 to 5) and attending physicians. Whenever a PCGS result was unclear, our physicians consulted a staff microbiologist, although this option was only available during daytime hours. Otherwise, they evaluated the PCGS results themselves.

In the laboratory, CSF samples were subjected to Gram stain, Ziehl–Neelsen stain, Indian ink stain, and cultures for the detection of etiologic agents. With regard to the Gram stain procedure, only the solutions were different from those usually used for PCGS. The laboratory technicians used Favor G manufactured by Nissui Pharmaceutical (Tokyo, Japan). This was a three-step staining procedure: 0.2% Victoria blue, 2% picric acid alcohol, and 0.04% fuchsine.

### PCGS and selection of antimicrobial agents for bacterial meningitis

In Gram staining of the CSF, Gram-positive diplococci or cocci in chains suggested *Streptococcus pneumoniae*, *Streptococcus* spp., *Enterococcus* spp., or other Gram-positives including *Lactococcus*. In such cases, a third-generation cephalosporin (cefotaxime or ceftriaxone) and vancomycin was frequently selected given the high proportion of penicillin-resistant *S. pneumoniae* of the CSF in Japan [24]. If there was a possibility of *Enterococcus* infection, ampicillin or penicillin G was combined with these agents. Gram-negative rods suggested Enterobacteriaceae such as *Klebsiella pneumoniae* or *Escherichia coli*, or other Gram-negatives including *Pseudomonas aeruginosa*; in such cases a third-generation cephalosporin (cefotaxime, ceftriaxone, or ceftazidime) was chosen. If an extended-spectrum beta-lactamase (ESBL)-producing bacterial strain was suspected based on previous culture findings, meropenem was selected. Gram-positive small rods suggested *Listeria monocytogenes*, in which case ampicillin was used. Gram-negative coccobacilli suggested *Haemophilus influenzae*. Gram-negative diplococci suggested *Neisseria meningitidis*.

In the simulated portion of our study, we reviewed all findings other than Gram stain findings from the same 34 bacterial meningitis patients and selected a course of treatment according to the current Japanese guidelines. When the guidelines recommended empiric antimicrobial therapy, we referred to the Practical Guideline for Bacterial Meningitis 2014 (PGBM), published by Societas Neurologica Japonica, the Japanese Society of Neurological Therapeutics and the Japanese Society for Neuroinfectious Diseases [24], to determine which antimicrobial agent would be used. If patients had debilitating associated diseases or immunodeficiency, the PGBM recommended ceftazidime (2 g q8h or 6 g/day) plus vancomycin (30-60 mg/kg/day or 3 g/day) plus ampicillin (2 g q4h or 12 g/day), or meropenem (2 g q8h or 6 g/day) plus vancomycin. If patients were immunocompetent and were over 50 years of age, a third-generation cephalosporin (cefotaxime 2 g q4-6 h or 8-12 g/day, or ceftriaxone 2 g q12h or 4 g/day) plus vancomycin plus ampicillin, or meropenem plus vancomycin, was recommended. Otherwise, the PGBM recommended meropenem.

For each bacterial meningitis patient, we compared the targeted therapy that was actually selected based on PCGS findings with the empirical therapy that would have been recommended by the PGBM in the simulation excluding PCGS findings. As part of this comparison, we analyzed the first-choice antimicrobials in terms of their dosage and cost, i.e., their original pharmaceutical price in Japanese yen, which is determined by the Ministry of Health, Labour and Welfare, as of the first day of admission.

### PCGS and rates of empiric antibiotic use in aseptic meningitis

To assess the utility of PCGS in selecting optimal treatments for aseptic meningitis patients, we compared the rate of empiric antibiotic use in our aseptic meningitis patients, all of whom were known to have negative Gram stain results, with the rate of antibiotic use in a previous study in which Gram staining findings were not available [11].

### Outcome measures

Primary outcomes were the sensitivity and specificity of PCGS for the differential diagnosis between bacterial and aseptic meningitis, the rate of meropenem administration among bacterial meningitis patients, and the rate of all antibiotic administration excluding antiviral agents among aseptic meningitis patients. Meropenem was selected for particular study because it has broad-spectrum coverage and is highly recommended as an empiric treatment by the Japanese guidelines as stated in the PGBM. Vancomycin is also broad-spectrum but is not suitable as an outcome measure because drug-resistant *S. pneumoniae* in the CSF is common in Japan, and the co-administration of vancomycin is necessary even if *S. pneumoniae* is suspected by PCGS. Secondary outcomes were the accordance between PCGS and CSF culture results; the effectiveness, as assessed by drug susceptibility tests on CSF cultures, of the antibiotics chosen based on PCGS findings; and the total drug cost on the first day of admission.

### Statistical analysis

To ensure an adequate sample size of bacterial meningitis patients, we initially planned to collect at least 30 patients, which would give us the minimum statistical power necessary to calculate sensitivity and specificity. We assumed that the rate of meropenem administration in the simulated PGBM-based portion of the study would be around 0.5, because the PGBM recommends the empiric use of meropenem or a third-generation cephalosporin for both immunocompromised and immunocompetent patients. In the PCGS group, in contrast, we expected a much lower rate of meropenem administration. Therefore, assuming that the rate of meropenem use would be 0.5 in the guideline group and 0.2 in the PCGS group, and assuming a guideline-group to PCGS-group ratio of 1:1, 80% power, and a one-sided alpha level of 0.05, we calculated that 31 patients per group would be needed.

Among aseptic meningitis patients, we hypothesized that a 20% difference in the rates of antibiotic administration between the PCGS and non-PCGS groups would be clinically meaningful. In a study in Switzerland where round-the-clock access to Gram staining was not available [11], the rate of empiric antimicrobial use in adults with aseptic meningitis was 0.6 (45/74). To detect a rate of empiric antibiotic use of 0.4 in the PCGS group, therefore, assuming 80% power and a one-sided alpha level of 0.05, we estimated that at least 79 patients would be needed.

The *χ*^2^ test or Fisher’s exact test was used for categorical variables, and Student’s *t*-test was used for numerical variables. The receiver operating characteristic (ROC) curve was analyzed to determine the best threshold for each variable to discriminate between bacterial and aseptic meningitis. The sensitivity, specificity, accuracy, positive and negative likelihood ratios, and areas under the ROC curve with 95% confidence intervals were calculated. Kappa statistics were also used to evaluate the agreement between the results of PCGS and those of CSF cultures. These results were calculated using Stata software (version 16.1; StataCorp, College Station, TX, USA).

## Results

Thirty-four patients with bacterial meningitis and 97 patients with aseptic meningitis were enrolled. Table 1 shows a comparison of bacterial and aseptic meningitis cases. Patient age ranged from 22 to 91 in bacterial meningitis cases and from 15 to 76 in aseptic meningitis cases.

**Table 1:** Comparison between bacterial and aseptic meningitis

	Bacterial meningitis		Aseptic meningitis		*p* value
	N = 34		N = 97		
Median age (IQR)	60 (44–68)		30 (23–34)		< 0.0001*
Male sex	19/34 (55.9%)		51/97 (52.6%)		0.74
Medical histories					
Symptom duration days (IQR)	2.5 (0–5)		3 (1–4)		0.71
Shaking chills	9/34 (26.5%)		6/97 (6.2%)		0.001*
Previous antibiotic exposure within 48 h	12/34 (35.3%)		9/97 (9.2%)		< 0.001*
Immunocompromised host	17/34 (50.0%)		8/97 (8.2%)		< 0.001*
Vital signs and physical examinations					
Systolic blood pressure (mmHg) (IQR)	136 (120–150)		112 (100–120)		< 0.0001*
Pulse rate (/min) (IQR)	113 (100–126)		88 (80–96)		< 0.0001*
Respiratory rate (/min) (IQR)	24 (20–30)		20 (18–22)		0.0002*
Body temperature (℃) (IQR)	38.6 (37.9–39.3)		37.7 (37.1–38.4)		< 0.0001*
Glasgow Coma Scale (IQR)	11 (9–14)		15 (15–15)		< 0.0001*
Neck stiffness	28/33 (84.9%)		47/95 (49.5%)		0.002*
Jolt accentuation	5/6 (83.3%)		53/64 (82.8%)		0.97
Laboratory data					
White blood cell counts (/μL) (IQR)	13,800 (11,000–16,200)		7800 (5900–9900)		< 0.0001*
C-reactive protein (mg/dL) (IQR)	6.7 (1.3–19.4)		0.18 (0.02–0.9)		< 0.0001*
CSF cell counts (/μL) (IQR)	1116 (140–4200)		80 (37–156)		< 0.0001*
CSF monocytes (%) (IQR)	5 (3–17)		74 (27–95)		< 0.0001*
CSF protein (mg/dL) (IQR)	287 (100–420)		66 (51–94)		< 0.0001*
CSF glucose (mg/dL) (IQR)	37 (1–59)		57 (53–63)		0.0001*
CSF glucose/blood glucose ratio (IQR)	0.17 (0.006–0.43)		0.55 (0.50–0.60)		< 0.0001*
CSF bacteria confirmed with PCGS	31/34 (91.2%)		1/97 (1.0%)		< 0.001*
CSF culture positivity	34/34 (100%)		0		< 0.001*
Blood culture positivity	29/34 (85.3%)		0		< 0.001*
Etiologic agent	*Streptococcus pneumoniae*	20	Mumps virus	8	
	*Klebsiella pneumoniae*	5	*Varicella zoster*	5	
	*Streptococcus bovis*	4	Cytomegalovirus	2	
	*Bacteroides fragilis*	1	*Herpes simplex*	2	
	*Escherichia coli*	1	Unknown	80	
	*Haemophilus influenzae*	1			
	*Lactococcus lactis cremoris*	1			
	*Listeria monocytogenes*	1			
Complications					
Infection sites other than meningitis	9/34 (26.7%)		13/97 (13.4%)		0.079
* Strongyloides* coinfection	10/34 (29.4%)		0		< 0.001*
Treatment					
Antibiotics other than acyclovir	34/34 (100%)		38/97 (39.2%)		< 0.001*
Steroid use	4/34 (11.8%)		0		0.005*
Antimicrobial treatment days (IQR)	14 (12–14)		3 (3–5)		0.0001*
Outcome					
Death	4/34 (11.8%)		0		0.004*
Neurological sequelae	4/30 (13.3%)		0		0.004*

*CSF* cerebrospinal fluid, *IQR* interquartile range, *PCGS* point-of-care Gram stain

* *p* < 0.05

In twelve patients with bacterial meningitis, antibiotics had previously been prescribed; these were amoxicillin, amoxicillin/clavulanic acid, piperacillin, cefotiam (2nd generation cephalosporin), cefmetazole, cefotaxime, ceftriaxone, cefoperazone/sulbactam, clarithromycin, ofloxacin and levofloxacin. No antiviral agents were prescribed. In nine patients with aseptic meningitis, penicillin G, amoxicillin/clavulanic acid, cefalexin, clarithromycin, fleroxacin, levofloxacin, acyclovir, or oseltamivir had been administered within 48 h prior to arrival.

There was only one patient with pneumococcal meningitis whose CSF cell count was 0. This patient’s CSF protein was not elevated (48 mg/dL) and CSF glucose was decreased (49 mg/dL) relative to blood sugar (127 mg/dL). Gram-positive diplococci were detected with PCGS.

*S. bovis* was reclassified into three different biotypes in 2003 [25]. Among the four S. *bovis* cases, only one could be more specifically identified as *Streptococcus infantarius* subsp. *coli*. The other three cases were of unidentified subtype because they were old cases from before the subtypes were defined. The five cases in which CSF cultures were positive but blood cultures were negative were two *S. pneumoniae*, one *H. influenzae*, one *K. pneumoniae*, and one *S. bovis* infection. Only one patient was infected with two bacterial species: *Bacteroides fragilis* was cultured from the CSF, and both *B. fragilis* and *E. coli* were cultured from the blood. No ESBL-producing bacteria were found in this study.

In the aseptic meningitis cases, mumps, *Varicella zoster*, *Herpes simplex*, and cytomegalovirus were identified as the etiologic agents either clinically or through serologic tests or polymerase chain reactions, but not through viral cultures.

Among the bacterial meningitis cases, the point of entry varied: four cases were suspected to have arisen through contiguous spread from local infection: these were three cases of otitis media and one case of sinusitis. The remaining seven cases were suspected to have emerged from distant foci of infection: these were three cases of pneumonia, two cases of septic arthritis, and one case each of infective endocarditis and osteomyelitis. Both septic arthritis cases were co-infected: one with infective endocarditis and one with osteomyelitis. Thus, nine patients had other simultaneous infections. Among the aseptic meningitis patients, thirteen cases had simultaneous other infections: eight of these were mumps in the parotid gland, four were skin lesions of *Varicella zoster*, and one was *Herpes simplex* virus in the genital area.

*Strongyloides stercoralis* coinfection was determined based on the presence of *S. stercoralis* larvae or eggs in the stool, gastric juice or sputum, or on previous episodes of proven strongyloidiasis associated with repeated meningitis. Ivermectin or thiabendazole was used with antimicrobials to treat *S. stercoralis* coinfection.

Among the 38 aseptic meningitis cases treated with empirical antimicrobial treatments, the antimicrobial agents were as follows: 19 ceftriaxone, 15 cefotaxime, 6 ampicillin, 3 vancomycin, and 1 doxycycline. Doxycycline was empirically chosen for possible leptospirosis, although the possibility of leptospirosis was later disproven. More than one antimicrobial was used simultaneously in several cases. In addition, acyclovir was empirically used in 10 patients for possible herpes infection.

Four bacterial meningitis patients survived with neurological sequelae; these included one with both sensory hearing loss and facial palsy, and one each with sensory hearing loss, neurogenic bladder, and impaired consciousness. In all of these, the etiologic agent was *S. pneumoniae*. Dexamethasone was administered in one case but not in the others.

Four patients died because of bacterial meningitis. In one of them, small Gram-negative rods were observed in PCGS, and ceftazidime and clindamycin were chosen accordingly. In this case, *Bacteroides fragilis* was cultured from the CSF, and both *B. fragilis* and *E. coli* were cultured from the blood.

Table 2 compares the effectiveness of PCGS with that of other variables for differentiating between bacterial and aseptic meningitis. Among the age and history variables, age over 45 had the highest area under the ROC curve (AUC). Among the vital signs and physical examination variables, Glasgow Coma Scale (GCS) less than 15 had the highest AUC. Among PCGS and laboratory data, PCGS had the highest AUC. Among all variables, PCGS was the most accurate predictor. When age was less than 45, GCS was 15, and PCGS was negative, 98.8% (84/85) of cases were aseptic meningitis.

**Table 2:** Effectiveness of different variables at correctly predicting a diagnosis of bacterial vs. aseptic meningitis

	Sensitivity	Specificity	Accuracy	LR +	LR−	AUC (95% CI)
Age and history						
Age≥45	73.5	89.7	85.5	7.1	0.30	0.82 (0.73–0.90)
Immunocompromised host	50.0	91.2	80.9	6.1	0.54	0.71 (0.62–0.80)
Shaking chills	26.5	93.8	76.3	4.3	0.78	0.60 (0.52–0.68)
Vital signs and physical examinations						
Glasgow Coma Scale < 15	81.2	97.9	93.7	38.2	0.19	0.90 (0.83–0.97)
Pulse rate≥100(/min)	76.4	80.0	79.1	3.8	0.29	0.78 (0.70–0.86)
Systolic BP≥120(mmHg)	85.3	61.1	67.4	2.2	0.24	0.73 (0.65–0.81)
Respiratory rate≥22(/min)	64.7	73.9	71.4	2.5	0.48	0.69 (0.60–0.79)
Neck stiffness	84.9	50.5	59.4	1.7	0.30	0.68 (0.60–0.76)
Body temperature≥38.0(℃)	69.7	61.1	63.3	1.8	0.50	0.65 (0.56–0.75)
PCGS and laboratory data						
CSF PCGS	91.2	99.0	97.0	88.4	0.09	0.95 (0.90–1.00)
CSF monocyte≤20(%)	85.3	83.3	83.9	5.1	0.18	0.84 (0.77–0.91)
CSF glucose/blood glucose ratio≤0.5	85.3	75.5	78.1	3.5	0.20	0.80 (0.73–0.88)
WBC≥10,000(/μL)	78.8	76.8	77.3	3.4	0.28	0.78 (0.70–0.86)
CSF protein≥100(mg/dL)	76.5	77.3	77.1	3.4	0.30	0.77 (0.69–0.85)
CRP≥1.0(mg/dL)	77.4	76.0	76.4	3.2	0.30	0.77 (0.68–0.85)
CSF cell≥100(/μL)	76.5	58.8	63.4	1.9	0.40	0.68 (0.59–0.76)

*AUC* area under curve, *BP* blood pressure, *CI* confidence interval, *CRP* C-reactive protein, *CSF* cerebrospinal fluid, *GCS* Glasgow Coma Scale, *LR* likelihood ratio, *PCGS* point-of-care Gram stain, *WBC* white blood cell

Table 3 shows the concordance between PCGS and CSF cultures. The kappa coefficient was 0.903 (95% Confidence Interval 0.887–0.906). The five cases whose PCGS results did not match their CSF culture results were one false-positive, three false-negatives, and one morphological mismatch. None of them had previous antimicrobial administration. In the one false-positive case, Gram-positive coccus was detected through PCGS but not cultured. The number of Gram-positive cocci in this case was low. Gram-positives were not confirmed through Gram stain in the laboratory. In one of the false-negatives, no bacteria were observed through PCGS, but a Gram-positive rod species identified as *L. monocytogenes* was cultured. This patient was an alcohol abuser. In another false-negative, no bacteria was observed through PCGS, but *S. pneumoniae* was cultured. This patient also had septic arthritis of the knee and infective endocarditis. In the third false-negative, no bacteria was confirmed through PCGS or Gram staining in the laboratory, but a Gram-negative rod identified as *H. influenzae* was cultured. The blood culture was also negative. This patient had a past medical history of CSF leakage. In the morphological mismatch case, Gram-negative coccobacilli were suspected through PCGS. However, a physician consulted the laboratory, and they revealed Gram-positive diplococci. This case was the second incidence of CSF leakage in a patient who had had *H. influenzae* infection previously.

**Table 3:** Concordance between point-of-care Gram stain and CSF culture results

	CSF culture			
	GPC	GNR	GPR	Negative
Point-of-care Gram stain				
GPC	23	0	0	1
GNR	1	7	0	0
GPR	0	0	0	0
Negative	1(Sp)	1(Hi)	1(Lm)	87

*CSF* cerebrospinal fluid, *GNR* Gram-negative rod, *GPC* Gram-positive coccus, *GPR* Gram-positive rod, *Hi* *Haemophilus influenzae*, *Lm* *Listeria monocytogenes*, *Sp* *Streptococcus pneumoniae*

Kappa coefficient 0.903 (95% CI 0.887–0.906)

Table 4 compares the targeted antimicrobial agent that was chosen based on PCGS results with the agent that would have been chosen in the same situation in the absence of PCGS data according to the current Japanese guidelines, i.e., the simulated empirical agent. When PCGS data was used, third-generation cephalosporins (cefotaxime and ceftriaxone) were used more frequently, and ceftazidime and meropenem were used less frequently, than they would have been in empirical treatment based on the Japanese guidelines. Meropenem was used in only one case in which Gram-negative rods were observed despite cefotaxime administration.

**Table 4:** Initial antibiotics selected based on PCGS versus those that would have been chosen under the Japanese guidelines

	Point-of-care Gram stain (N = 34)				Japanese guidelines (N = 34)				*p* value
	GPC (N = 23)	GNR (N = 8)	False-negative (N = 3)	Total	Immunocompetent		Immunocompromised (N = 17)	Total	
					Age < 50 (N = 7)	Age≥50 (N = 10)			
Ampicillin/Penicillin G	10/1	4/0	1/0	16	0	5/0	8/0	13	0.62
Cefotaxime/Ceftriaxone	17/6	5/1	3/0	32	0	3/2	0	5	< 0.001*
Ceftazidime	0	1	0	1	0	0	8	8	0.027*
Vancomycin	18	1	2	21	0	10	17	27	0.18
Meropenem	0	1	0	1	7	5	7	19	< 0.001*
Clindamycin	0	1	0	1	0	0	0	0	1.000

*GNR* Gram-negative rod, *GPC* Gram-positive coccus, *PCGS* point-of-care Gram stain

*p < 0.05

Drug susceptibility tests of CSF cultures revealed that all antimicrobial treatments that were chosen based on PCGS results were effective against bacterial meningitis. All treatments that would have been chosen in the absence of PCGS findings were also effective.

The median dose (interquartile range) on the first day of admission for each antimicrobial chosen based on PCGS was as follows: ampicillin 8.5 g (8-12 g), penicillin G 24 million units (24–24 million units), cefotaxime 9 g (8-12 g), ceftriaxone 4 g (4-4 g), vancomycin 2 g (2-2 g), meropenem 6 g (6-6 g). The total cost on the first day of admission for all antimicrobials chosen based on PCGS was 78.5% of the total cost of all antimicrobials that would have been chosen under the Japanese guidelines (416,353 vs. 530,091 Japanese yen).

In aseptic meningitis, 39.2% (38/97) of patients were treated with empiric antibiotics despite having negative PCGS findings (Table 1). This proportion was lower than that in a previous study on adult aseptic meningitis in which Gram staining was not available (60.8%, 45/74) (p = 0.006) [11].

## Discussion

This observational study investigated the usefulness of PCGS in diagnosing and treating both bacterial and aseptic meningitis in adults. Our results yielded three main findings. First, PCGS was the most precise predictor for differentiating between bacterial and aseptic meningitis, being superior to medical histories, vital signs and physical examinations, and laboratory data available in the ER. Second, for bacterial meningitis, the selection of targeted therapies based on PCGS resulted in less frequent use of broad-spectrum antimicrobials compared with the simulated selection of empirical therapies based on the Japanese guidelines reported in the PGBM. Third, for aseptic meningitis, the selection of empiric therapies based on PCGS resulted in less frequent use of unnecessary antibiotics compared with a previous study in which Gram stain was not available.

Although most PCGS examinations in this study were performed by trainee doctors, the sensitivity and specificity of PCGS for detecting bacteria were 91.2% and 99.0% in bacterial and aseptic meningitis, respectively. Identification of bacterial strains based on PCGS was strongly correlated with that based on CSF culture results. These results are comparable to those of previous studies on Gram staining in microbiological laboratories [9, 12–14]. Accurate interpretation of CSF Gram stain results largely depends on the expertise of the individual performing the test [26]. PCGS can be interpreted with high accuracy by doctors who are experienced at performing and reading Gram stains, as studies on respiratory and urinary tract infections have shown [18, 27, 28]. At Okinawa Chubu Hospital, trainee doctors are still taught to consider Gram stain as part of a normal physical examination, as doctors in the United States used to be [23, 29, 30]. Their extensive experience with Gram stain and their eagerness to obtain PCGS results as soon as possible in order to initiate optimal antimicrobials enables them to perform and interpret PCGS with speed and accuracy.

In the sole false-positive case, a scant quantity of Gram-positive cocci was observed through PCGS, but this strain did not incubate in CSF culture. No previous antimicrobial agent had been administered in this case. Thus, debris or artifacts were suspected to have contaminated the Gram stain solution.

With regard to the three false-negative cases, one was *L. monocytogenes*, an intracellular pathogen for which Gram staining offers a particularly low sensitivity of less than 50% [21, 31]. In the second case, the meningitis was a secondary infection site, while the primary infection focus was infective endocarditis or septic arthritis. The third case was an *H. influenzae* infection that had spread through CSF leakage. Previous studies have shown that the accuracy of Gram stain, that is, the correspondence between Gram staining and CSF culture results, depends strongly on bacterial load: Gram stain results were positive in only 25% of meningitis cases in which the CSF contained less than 10^3^ CFU/ml, in 60% of cases with 10^3^ to 10^5^ CFU/ml, and in 97% of cases with more than 10^5^ CFU/ml [10, 32]. Therefore, it is likely that, in each of our three false-negative cases, the bacterial load in the CSF was too small for PCGS to detect the bacteria. In spite of these cases’ negative PCGS results, empirical antimicrobials were started in all three because bacterial meningitis was suspected due to low glucose level and high protein level in the CSF, strong suspicion of infectious endocarditis, and history of CSF leakage in the three cases, respectively.

In the one morphological mismatch case, PCGS seemed to indicate Gram-negative coccobacilli, but Gram stain in the laboratory revealed Gram-positive diplococci. After incubation, *S. pneumoniae* were identified through CSF culture, and the result of the Gram stain in the laboratory was correct. The reason of this mismatch could be that the crystal violet in the first stain was decolorized too much with acetone ethyl alcohol during the PCGS procedure and misidentified as Gram-negative. In the laboratory, Victoria blue was used for the first stain, and the blue was so vivid that it was easy to recognize Gram-positive cases. An obvious mismatch between PCGS and laboratory-performed Gram stain was not found in the other cases we reviewed.

Compared with the simulated empirical therapies based on the Japanese guidelines reported in the PGBM, the targeted therapies based on PCGS were narrower-spectrum, equally effective, and 20% lower in cost. PCGS enabled us not only to detect the presence of bacteria but also to identify their morphology. This permitted us to choose narrower-spectrum antimicrobials than the PGBM recommended, allowing us to keep pharmaceutical costs lower [18, 33]. If Gram-positive diplococcus was detected in PCGS, both third-generation cephalosporin and vancomycin were recommended because of the high prevalence of penicillin-resistant *S. pneumoniae* infection of the CSF in Japan [24]. The nationwide rate of *S. pneumoniae* resistance to penicillin-G in bacterial meningitis as assessed through CSF culture was 68.8% (121/176) in 2001–2008 [34]. For pneumococcal meningitis, however, the co-administration of penicillin or meropenem was deemed unnecessary in the present clinical setting because the spectrum of third-generation cephalosporin with vancomycin sufficiently covers all of *S. pneumoniae* including the penicillin-resistant strains. If a Gram-negative rod was detected, vancomycin, which covers only Gram-positive species, was deemed unnecessary. The decision of whether to use carbapenem depended on local drug resistance patterns. In this population, meropenem was used in only one case, and ESBLs were not detected. It is expected, however, that meropenem use will increase in the future, given the ongoing increase in the incidence of ESBL-producing Enterobacteriaceae [24].

In aseptic meningitis, empiric antibiotics were used less frequently in our study, where PCGS findings were available, than they were in another study where Gram staining was only available during daytime hours [11]. The proportion of aseptic meningitis cases treated with empiric antimicrobials varies among institutions. A study in France reported using antimicrobials in only 35.2% of aseptic meningitis cases (19/54), although this figure excludes cases with previous antimicrobial exposure [3]. A study in the USA, in contrast, reported using antimicrobials in 73.5% of aseptic meningitis cases (297/404), although this figure includes cases with previous antimicrobial exposure [35]. Another reason why it is difficult to compare our rate of antimicrobial use for aseptic meningitis with those in other regions is that the etiology of aseptic meningitis may vary among populations. However, the rate of empiric antimicrobial use after Gram stain results obtained later was 47.2% (35/74) [11], and it was similar to that at our institution (*p* = 0.28). Therefore, using PCGS anytime could help avoid the overuse of empiric antibiotics even for aseptic meningitis.

In bacterial meningitis cases, steroids were used in only 4/34 cases (11.8%). There were two reasons for this. One was that the study period was between 1995 and 2015. It was only in 2002 that the early use of dexamethasone for bacterial meningitis was proven to be effective, especially in patients with pneumococcal meningitis with GCS 8 to 11 [36]. The other was that some of our bacterial meningitis cases (10/34) were caused by *S. stercoralis* hyperinfection in this area [37], and steroid therapy is known to be harmful in some cases of strongyloidiasis [37]. Confirming the presence of Gram-negative rods or Gram-positive cocci in chains encouraged us to search for *S. stercoralis* and to consider the possibility of disseminated strongyloidiasis.

This study has several limitations. First, some selection bias exists. The bacterial meningitis cases included only cases with positive CSF cultures. We excluded cases of meningitis related to surgical intervention, encephalitis, and probable aseptic meningitis with antimicrobial exposure in the previous 48 h. HIV-infected patients were also excluded. In practice, however, hospitals will see many cases in which antimicrobials have already been administered, preventing clinicians from distinguishing between bacterial and aseptic meningitis. Second, this is a retrospective chart review study. We compared the accuracy of PCGS with that of Gram staining performed at a microbiological laboratory to the extent possible, though some old data were missing. Third, our results may not be directly applicable to other hospitals where PCGS is not regularly performed or where the prevalence of the various etiologic agents of meningitis is different. At most other hospitals, clinicians are unfamiliar with PCGS and lack the experience required to read Gram stain results [11]. Fourth, quality control of PCGS was not assessed in this study, although PCGS is the standard of care at Okinawa Chubu Hospital and its quality has been confirmed in several other studies [18, 27, 28]. Fifth, our ability to confirm the etiologies of aseptic meningitis was limited because further examinations other than those on the commercial level were not performed.

## Conclusions

PCGS of CSF by clinicians is a valuable diagnostic tool for the discrimination of bacterial and aseptic meningitis in adults. PCGS enables the selection of narrower-spectrum antimicrobials for bacterial meningitis compared with the Japanese guidelines, and can potentially reduce the rate of empiric antimicrobial therapies for aseptic meningitis.

## Data Availability

The datasets used and analyzed during the current study are available from the corresponding author on reasonable request.

## Abbreviations

AUC: Area under the ROC curve
CSF: Cerebrospinal fluid
ER: Emergency room
ESBL: Extended-spectrum beta-lactamase
GCS: Glasgow Coma Scale
HIV: Human immunodeficiency virus
HTLV-1: Human T-lymphotropic virus-1
PCGS: Point-of-care Gram stain
PGBM: Practical Guideline for Bacterial Meningitis 2014
ROC: Receiver operating characteristic
